# University of Maryland Dental Department

**Published:** 1883-11

**Authors:** 


### Editorial, Etc.

University of Maryland Dental Department.—The many friends of this institution will be pleased to learn that the number of students attending the present Session of 1883-84 has exceeded all expectations. At the present time of writing October 31st, the number of matriculates is far in excess of the entire number during the last session. The Infirmary Practice is equal to all demands, and is rapidly ncreasing to such a degree, that what was formally considered to be ample space for its accommodations has proven inadequate, and will necessitate the erection of a large addition to the .present Infirmary and Laborarory building. Not only number but character, intelligence and deportment, distinguish the present class, many of whom are sent to this institution by some of the most reputable practicing dentists of this country and Europe.

